# Parent Gender Affects the Influence of Parent Emotional Eating and Feeding Practices on Child Emotional Eating

**DOI:** 10.3389/fpsyg.2021.654237

**Published:** 2021-09-10

**Authors:** Shaina D. Trevino, Nichole R. Kelly, Elizabeth L. Budd, Nicole R. Giuliani

**Affiliations:** ^1^Department of Counseling Psychology and Human Services, Prevention Science Institute, University of Oregon, Eugene, OR, United States; ^2^Department of Special Education and Clinical Sciences, Prevention Science Institute, University of Oregon, Eugene, OR, United States

**Keywords:** parent emotional eating, child emotional eating, feeding practices, child eating behavior, parental feeding, parent gender

## Abstract

Extant research supports a direct association between parent’s own emotional eating and their child’s emotional eating, and demonstrates correlations among parent emotional eating, feeding practices, and child emotional eating. However, the majority of this work focuses on the separate influences of these factors. The current study aims to add to the literature by simultaneously examining the indirect effects of three major parental feeding practices (i.e., emotion regulation, instrumental, and restrictive feeding) in the association between parent emotional eating and child emotional eating, and exploring how these indirect effects vary based on parent gender. Parents (86 fathers, 324 mothers) of an elementary school-age child (*M* = 8.35, *SD* = 2.29, range = 5–13) completed an online survey through Qualtrics Panels. Results suggested that restrictive feeding partially accounted for the association between parent and child emotional eating in the combined sample of mothers and fathers. Exploratory analyses revealed that the indirect effects of parental feeding practices in the association between parent emotional eating and child emotional eating varied based on parent gender. Among mothers, restrictive feeding was the only feeding practice that partially accounted for the association between maternal and child emotional eating, whereas all three feeding practices fully accounted for the association between father and child emotional eating. As the bulk of the literature on parent emotional eating and feeding has solely focused on mothers, these findings offer insight into how feeding practices may differentially function in the relation between parent emotional eating and child emotional eating for mothers versus fathers.

## Introduction

### Background

Emotional eating is characterized by eating in response to emotions instead of internal hunger cues (Bruch, 1964). Emotional eating is associated with overeating (van Strien et al., 2005), as well as other disordered eating behaviors, including bulimia and binge eating (Ashcroft et al., 2008). Emotional eating is also positively related to disordered eating attitudes such as, weight concern (Barnhart et al., 2021) and body dissatisfaction (Annesi and Mareno, 2015). Both children (Braet and van Strien, 1997; Webber et al., 2008) and adults (van Strien et al., 2009) who engage in emotional eating are more likely to have a higher body mass index (BMI; usually defined as equal to or over 25), which is a risk factor for chronic diseases (e.g., diabetes; Boles et al., 2017). Further, childhood emotional eating has been shown to predict disordered eating behaviors (e.g., binge eating; Allen et al., 2008) and increased adiposity up to 4 years later (Shriver et al., 2019). While emotional eating can occur in response to both positively and negatively valenced emotions, the majority of the research in this area defines emotional eating as using food to escape or reduce negative emotions (e.g., Arnow et al., 1995). To maintain consistency with the literature, this study focuses on eating in response to negative emotions.

Research suggests that the rates of emotional eating are lower in young children, but increase during middle childhood and adolescence (e.g., Wardle et al., 2001; van Strien and Oosterveld, 2008), with one study reporting that 63% of children ages 5–13 have engaged in emotional eating in their lifetime (Shapiro et al., 2007). The bulk of the research on how children adopt emotional eating practices has focused on the role of mothers and their preschool-aged children (e.g., Wardle et al., 2002; Rodgers et al., 2014). In middle childhood, children often have much more autonomy and less dependence on parents, especially regarding food access (Steinsbekk et al., 2018). Extant work has identified parent eating and feeding practices as correlates of children’s emotional eating in middle childhood (Braden et al., 2014), but no study to date has directly disentangled parent eating, feeding, and child eating across different primary caregivers in middle childhood.

### Parent and Child Emotional Eating

Some researchers have suggested that child emotional eating is a learned response (van Strien and Oosterveld, 2008; Blissett et al., 2010). Since parents are one of the primary socialization agents for their children, it is not surprising that parenting behaviors are related to child emotional eating. According to psychosomatic theory, child emotional eating is a maladaptive response to negative emotion that results from interactions with caregivers (Bruch, 1973). These caregivers also have a major role in shaping eating behaviors and determining eating environments (e.g., Birch and Fisher, 1998; Anderson and Butcher, 2006; Lindsay et al., 2006; Anzman et al., 2010; Sleddens et al., 2011).

Parents are not only the main providers of food for their child, but they also model eating behaviors (e.g., through their own emotional eating), and affect their child’s eating through parenting behaviors such as feeding practices (Ventura and Birch, 2008). For example, numerous studies document a direct association between parent emotional eating and their child’s emotional eating (e.g., Snoek et al., 2007; Jahnke and Warschburger, 2008; de Lauzon-Guillain et al., 2009; Elfhag et al., 2010; Kröller et al., 2013; Rodgers et al., 2014). This effect has mostly been explained through modeling: children observe parents eating in response to emotional stimuli and thus may be more likely to do the same when faced with emotional cues (Morrison et al., 2013). Parental feeding behaviors are also consistently associated with children’s emotional eating (e.g., Birch and Davison, 2001; Blissett et al., 2010; Rodgers et al., 2013; Braden et al., 2014; Farrow et al., 2015). Although parents are usually well-intentioned, their feeding behaviors can disrupt the child’s ability to regulate how they eat in response to physical hunger and satiety cues and lead to unhealthy eating behaviors, including emotional eating (Heatherton and Baumeister, 1991; Birch and Fisher, 1998).

### Parental Feeding Practices

Parental feeding practices can also be influenced by parents’ own eating behaviors (Sleddens et al., 2010). In a study of parents from the United States and France, researchers found that parent emotional eating behavior was associated with more use of instrumental feeding (de Lauzon-Guillain et al., 2009). However, the majority of this work has been focused on maternal eating and feeding practices. Mothers who engage in emotional eating have been found to report greater use of instrumental (Kröller et al., 2013) and emotional feeding practices (Wardle et al., 2002). As described above, such feeding practices often result in higher levels of child emotional eating. Here, we review the evidence for three types of parental feeding practices that have been linked to child emotional eating: emotion regulation feeding, instrumental feeding, and restrictive feeding.

#### Emotion Regulation Feeding

Emotion regulation feeding often occurs when parents respond to their child’s negative feelings (e.g., boredom, distress) by giving them food (Musher-Eizenman and Holub, 2007). Psychosomatic theory posits that child emotional eating results from children learning to use food to regulate negative emotions when parents repeatedly feed their children while they are upset (Bruch, 1964). Thus, parents who use emotion regulation feeding may be indirectly teaching their children to eat for comfort. Parent emotion regulation feeding has been associated with child emotional eating in numerous cross-sectional (e.g., Jahnke and Warschburger, 2008; Steinsbekk et al., 2018), longitudinal (Rodgers et al., 2013), and experimental studies (Blissett et al., 2010). However, the vast majority of these studies have investigated these processes in preschool-aged children. The few studies that exist on older children do support an association between emotion regulation feeding and emotional eating in children aged 5–12 (see Braden et al., 2014; Tan and Holub, 2015). Because of the increased food autonomy experienced in middle childhood (i.e., the elementary school years), additional investigations as to how parental eating and feeding function to influence child emotional eating in this age range is needed.

#### Instrumental Feeding

Instrumental feeding occurs when parents use food to regulate their child’s desired behavior (i.e., using food as a reward or punishment; Benton, 2004). Similar to emotion regulation feeding, using instrumental feeding can implicitly teach children that food can be used in response to non-nutritional needs (Evers et al., 2010), and inhibit their ability to self-regulate their eating (Musher-Eizenman et al., 2009). Over time, children learn to associate food with external stimulation, such as celebration, rather than hunger. Instrumental feeding is also related to child emotional eating (e.g., Rodgers et al., 2013, 2014; Powell et al., 2017). In particular, longitudinal studies have demonstrated that parent’s use of instrumental feeding practices at ages 5–7 significantly predicts child emotional eating (Steinsbekk et al., 2016), and eating in the absence of hunger when experiencing heightened stress (Farrow et al., 2015) several years later.

#### Restrictive Feeding

Restrictive feeding is a type of controlling feeding practice that occurs when parents limit their child’s access to and consumption of food (Fisher and Birch, 1999). Parents may restrict their child’s food intake for various reasons, such as restricting food for health or weight reasons (Musher-Eizenman and Holub, 2007), however, much of the research on emotional eating and restrictive feeding has disregarded different motivations for restrictive feeding in studies and focuses on frequency of this behavior instead. Although parents often restrict high-calorie foods, data show that children actually consume more of these foods when access is gained after they have been restricted (Fisher and Birch, 1999). Similar to emotion regulation and instrumental feeding, repeated restrictive feeding may disrupt a child’s internal hunger and satiety cues, which in turn can inhibit the child’s ability to self-regulate their food consumption (Faith et al., 2004; Jansen et al., 2012). Although there are studies that document a positive association between restrictive feeding and child emotional eating (e.g., Joyce and Zimmer-Gembeck, 2009; Kröller et al., 2013; Williams et al., 2017), other researchers have discovered no significant associations (Blissett et al., 2010). Thus, the relation between restrictive feeding and child emotional eating is less clear compared to emotion regulation or instrumental feeding.

### The Role of Parental Feeding in the Relation Between Parent and Child Emotional Eating

The research described above presents meaningful associations among parent emotional eating, feeding practices, and child emotional eating. However, the existing literature in this area suffers from two notable limitations. First, it largely focuses on the separate influences of parents’ eating and feeding practices on child emotional eating rather than their simultaneous impact. Thus, it is not clear which feeding practices have the strongest influence in the direct association between parent and child emotional eating. There are only a handful of studies that have compared the indirect effects of various parental feeding practices on the association between parent and child emotional eating. Of the two studies found, one study did not find significant indirect effects of maternal feeding practices in the association between mother-child emotional eating, but did not assess emotion regulation feeding (Kröller et al., 2013). Another study documented significant indirect effects of emotion regulation and instrumental feeding, but excluded restrictive feeding (Rodgers et al., 2014). The present study seeks to reproduce the latter finding and directly compare the indirect effects of emotion regulation feeding, instrumental feeding, *and* restrictive feeding in the relation between parent and child emotional eating in the same model.

Second, the vast majority of work on feeding practices affecting parent-child emotional eating has focused on mothers, despite the fact that evidence exists to suggest that feeding styles vary by parent gender. Studies have documented that, compared to mothers, fathers use more restrictive (Musher-Eizenman et al., 2007) and instrumental (Powell et al., 2017) feeding practices, and report feeling less responsible for feeding (Blissett et al., 2006). Further, the way children respond to parental eating and feeding styles can differ by parent gender (e.g., Johannsen et al., 2006). For example, one study found that child emotional eating is more influenced by emotional eating of mothers than fathers (Snoek et al., 2007). Others have discovered that maternal feeding practices result in higher levels of emotional overeating, whereas paternal feeding practices are more likely to lead to emotional undereating (Haycraft and Blissett, 2012; Wei et al., 2018). Although fathers are increasingly becoming more involved in feeding their children (see Khandpur et al., 2014; Mallan et al., 2014), the overwhelming majority of the extant work on how parent emotional eating and feeding practices affect child emotional eating has focused solely on mothers (e.g., Kröller et al., 2013; Rodgers et al., 2014). While a sizeable body of literature has examined how *child* gender affects child emotional eating and the feeding practices of mothers (e.g., Snoek et al., 2007; van Strien and Bazelier, 2007; Hoffmann et al., 2016), there is limited work on the influence of *parent gender* on the associations among parent emotional eating, feeding practices, and child emotional eating. It is not yet understood how fathers’ feeding practices and own emotional eating concurrently influence their child’s emotional eating or how these processes differ based on parent gender.

### Current Study

Given these gaps in the literature, the purpose of the current study is twofold. First, we aim to simultaneously examine how different parental feeding practices account for variance in the association between parent emotional eating and child emotional eating during middle childhood. Specifically, we will directly compare the indirect effects of parent’s use of instrumental, emotion regulation, and restrictive feeding practices, through which parent emotional eating relates to child emotional eating. This is a major gap in the field as many studies have examined only simple associations among these variables instead of a comprehensive model. Second, we aim to add to the literature on parental influences on child emotional eating by exploring how these indirect effects differ based on parent gender as existing research suggests mothers and fathers influence their child’s behavior differently. The results of this aim will also inform future interventions or recommendations for parents related to child emotional eating by revealing differences in parent emotional eating and feeding patterns, or lack thereof. These aims will be accomplished by testing a parallel indirect effects model and comparing a multiple group parallel indirect effects model, respectively.

## Materials and Methods

### Sample and Design

This study is part of a larger investigation on parent and child health behaviors and the home and school environments. The sample included independent families in which only one parent (e.g., mother or father) was recruited for each child. Parents (*N* = 410; 79% female) were recruited using an online market research sample aggregator (Qualtrics Panels; see Budd et al., 2020 for more details). To be eligible to participate, individuals had to live in Oregon, have an elementary school-age child, and be able to read in English. Most participants identified as non-Hispanic White (80%), followed by more than one ethnicity (10%), Hispanic (3%), Asian (3%), other (1%), Black (1%), Native American/Alaska Native (<1%), Middle Eastern (<1%), and Pacific Islander (<1%). All other demographics are presented in Table 1. Children (49% female) were between the ages of 5–13 years-old (*M* = 8.4 years, *SD* = 2.29). This was a cross-sectional study and all responses were parent-report.

**TABLE 1:** Parent and child demographics by parent gender.

Variable	Fathers (21%) *n* = 86	Mothers (79%) *n* = 324	Full sample *N* = 410
	*n (%)/M (SD)*	*N (%)/M (SD)*	*N (%)/M (SD)*
**Parent demographics**			
Age (years)*			
18–29	7 (8%)	74 (23%)	81 (20%)
30–39	45 (52%)	162 (50%)	207 (50%)
40–49	20 (23%)	64 (20%)	84 (20%)
50–59	9 (10%)	17 (5%)	26 (6%)
Over 60	5 (6%)	7 (2%)	12 (3%)
**Child demographics**			
Female	35 (41%)	164 (51%)	199 (49%)
Age	8.79 (2.36)*	8.23 (2.26)*	8.35 (2.29)

*n/N, number of observations; *M*, mean; *SD*, standard deviation.*

*significantly different (*p* < 0.05).

### Measures

Parent and child emotional eating were assessed via the self and child emotional eating subscales, respectively, of the Dutch Eating Behavior Questionnaire (DEBQ; van Strien et al., 1986). Both self and child emotional eating subscales included 13 items, assessing eating in response to negative emotions, with a 5-point Likert-type response scale ranging from 1 (seldom) to 5 (very often). Sample items included: “Do you have a desire to eat when you are depressed or discouraged?” and “Do you have a desire to eat when you are feeling lonely?” Item scores were averaged for each subscale to create an average index of frequency of parent and child emotional eating behaviors. Higher scores indicated greater parent-reported emotional eating. Internal consistency was high for parent and child scales; Cronbach’s alphas (α) were 0.95 and 0.96, and McDonald’s omegas (ω) were 0.95 and 0.96, respectively, for parent and child scales.

Parental feeding practices were assessed with the Comprehensive Feeding Practices Questionnaire (CFPQ; Musher-Eizenman and Holub, 2007). The following subscales were included: (1) emotion regulation (3 items); (2) instrumental (3 items); and (3) restriction for weight control (8 items). Parents responded to statements about feeding practices on a 5-point Likert-type scale from 1 (never) to 5 (always). Item scores were summed to create a total index for each subscale. Higher scores indicated greater self-reported use of that feeding practice. Internal reliability for emotion regulation (α = 0.81, ω = 0.81), instrumental (α = 0.70, ω = 0.70), and restrictive (α = 0.86, ω = 0.86) subscales were adequate.

Parent gender was asked directly to participants of the survey (i.e., “With which gender do you identify?”). There were three response options (male, female, and other), although no parents selected “other.”

Parent stress, parent negative affect, child gender, and child age were included as covariates. These covariates were selected because previous research suggests they may contribute to variance in the statistical model. Specifically, studies have documented significant associations between child emotional eating and child gender (Braden et al., 2014) and maternal stress (Rodgers et al., 2014), as well as indirect associations with maternal negative affect (Rodgers et al., 2014). Covariates were also included if previous indirect models of parent-child emotional eating included them as controls, such as for child age (e.g., Kröller et al., 2013; Tan and Holub, 2015). Parent stress was assessed with a single subjective rating of stress (i.e., “Overall, how stressed are you?”) on a scale from 0 (not stressed at all) to 100 (extremely stressed) in the past month. Parent negative affect was assessed via the trait negative affect subscale on the Positive and Negative Affect Schedule (PANAS; Watson et al., 1988). Responses on the 10-item subscale range from 1 (not at all) to 5 (extremely). A summed score was used for analysis, and internal consistency was good (α = 0.86, ω = 0.86). Child age was chosen from a list (options: 5 through 13 years) and provided in integers. Child gender was assessed with a single item (i.e., “What is the gender of your child?”) with three response options (male, female, other), although no parents responded “other.”

### Analytic Strategy

Reliability metrics, such as Cronbach’s alphas were calculated with SPSS version 26, and McDonald’s omegas were computed with the OMEGA SPSS macro (Hayes and Coutts, 2020). While Cronbach’s alpha is a widely reported measure of internal consistency, McDonald’s omega requires fewer statistical assumptions that are difficult to meet (e.g., essential tau-equivalence) and has been recommended as an alternative (see Dunn et al., 2014; Hayes and Coutts, 2020), so both were reported. All subsequent analyses were conducted in R version 4.0.3 (R Core Team, 2019). Study variables were assessed for skew and kurtosis; variables with a skewness or kurtosis over −/+1 were transformed to improve distributions and re-assessed. All feeding practices (i.e., emotion regulation, instrumental, and restrictive) were identified as non-normally distributed. The distributions of these variables were greatly improved by transformation using the transformTukey function in the R package *rcompanion* package (Mangiafico, 2019), which follows the Tukey’s Ladder of Powers principle to improve the distribution of skewed variables. The transformed parental feeding variables were used for all statistical analyses and are reported in corresponding tables/figures.

**FIGURE 1:**
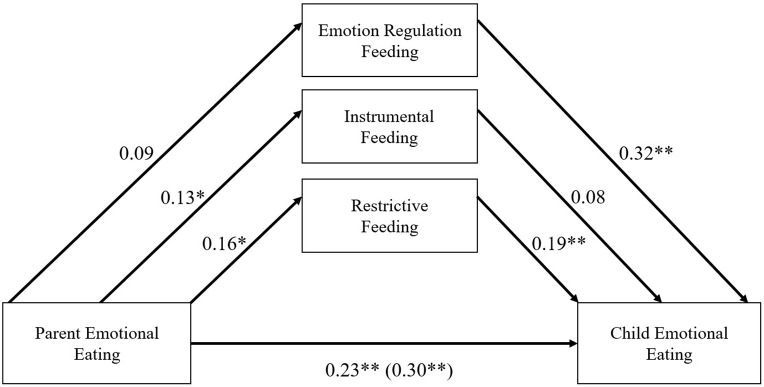
Standardized regression estimates for parallel indirect effects path model with the full sample. *p < 0.05, **p < 0.001. Covariates included parent stress, parent negative affect, child age, and child gender on all model paths.

A comparison between fathers and mothers on study variables were analyzed with Welch’s *t*-test for continuous variables, and Pearson’s chi-squared test for count variables in R. Path models were tested with a series of structural equation models (SEM) with the *lavaan* R package (Rosseel, 2012). There were two participants who did not answer one of the items for the DEBQ. Since both participants had zero variance in their ratings of the remaining items from that scale, their composite scores were calculated using the available items. Thus, there were no missing data in the composite variables that were included in the analysis. All models were tested with full information maximum likelihood estimation with robust standard errors, included all covariates (i.e., parent stress, negative affect, child age, and gender) for all model paths, and specified correlations among all feeding practices. When estimating indirect effects, bias-corrected bootstrapped standard errors and confidence intervals were estimated with the *RMediation* package (Tofighi and MacKinnon, 2011) and 10,000 resamples following the guidelines of MacKinnon (2008) to detect significant indirect effects. To test the indirect effect of the three parental feeding practices (i.e., emotion regulation, instrumental, and restrictive feeding) between parent and child emotional eating, all parental feeding practices were modeled as parallel indirect effects in addition to the direct effect of parent emotional eating to child emotional eating (see Figure 1). This allows for the simultaneous evaluation of each parental feeding practice while also accounting for the associations of all feeding practices (Hayes and Rockwood, 2017).

To explore how parent gender moderated the indirect effect of parental feeding practices between parent and child emotional eating, a multiple group structural equation analysis was conducted (see Kline, 2016). In the first step, path coefficients from the above model were freely estimated for both mothers and fathers. Then, path coefficients were constrained to be equal across groups. In the last step, path coefficients and intercepts were constrained to be equal across groups. Tests of model comparisons were then conducted to evaluate which model it the data significantly better. Comparative model fit was assessed with a chi-square difference test. Moderation was determined if the model with freely estimated group parameters fit the data significantly better than the models with constrained parameters. The parallel mediation model was just-identified, therefore, no overall model fit indices were calculated. All reported coefficients are standardized unless otherwise stated, and confidence intervals are reported at the 95% level. The dataset and analysis script are available online - https://osf.io/muzbj/.

## Results

### Preliminary Analyses

Descriptive statistics for study variables are presented in Table 2. Preliminary results from the Welch’s *t*-test showed that, compared to mothers (*M* = 1.80, *SD* = 0.65), fathers (*M* = 2.04, *SD* = 0.87) reported significantly greater use of instrumental feeding practices *t*(122) = −2.34, *p* < 0.05. Fathers (*M* = 1.94, *SD* = 0.84) also reported significantly greater use of restrictive feeding practices compared to mothers (*M* = 1.56, *SD* = 0.58), *t*(125) = −4.13, *p* < 0.05. Mothers (*M* = 57.40, *SD* = 23.67) reported significantly higher levels of stress than fathers (*M* = 50.74, *SD* = 25.69), *t*(126) = 2.17, *p* < 0.05. Regarding demographics, fathers (*M* = 8.79, *SD* = 2.36) had slightly older children compared to mothers (*M* = 8.23, *SD* = 2.26), and this difference was statistically significant, *t*(130) = −1.98, *p* < 0.05. The results of Pearson’s chi-square test revealed that there were significantly more fathers in the older age range categories than was expected if they were evenly distributed across ages for mothers and fathers, χ^2^ (4, *N* = 410) = 13.93, *p* < 0.05. There were no statistically significant differences between fathers and mothers in the remaining study variables (i.e., parent or child emotional eating, emotion regulation feeding, parent negative affect, and child gender).

**TABLE 2:** Descriptive statistics for study variables by parent gender.

Variable	Fathers (21%) *n* = 86	Mothers (79%) *n* = 324	Full sample *N* = 410	
	*M (SD)*	*M (SD)*	*M (SD)*	*Range*
Parent emotional eating (DEBQ self-report)	2.46 (0.92)	2.53 (0.80)	2.52 (0.83)	1–5
Child emotional eating (DEBQ parent-report)	1.94 (0.82)	1.93 (0.77)	1.94 (0.78)	1–4.7
Emotion regulation feeding (CFPQ)	1.66 (0.89)	1.47 (0.53)	1.51 (0.63)	1–5
Instrumental feeding (CFPQ)	2.04 (0.87)*	1.80 (0.65)*	1.85 (0.71)	1–5
Restrictive feeding (CFPQ)	1.94 (0.84)*	1.56 (0.58)*	1.64 (0.66)	1–5
Stress (single-item)	50.74 (25.69)*	57.40 (23.67)*	56.01 (24.23)	0–100
Negative affect (PANAS)	2.21 (0.90)	2.22 (0.77)	2.22 (0.80)	1–5

*n/N, number of observations; *M*, mean; *SD*, standard deviation; DEBQ, Dutch Eating Behavior Questionnaire; CFPQ, Comprehensive Feeding Practices Questionnaire; PANAS, Positive and Negative Affect Schedule. Range refers to the minimum and maximum observed values.*

*significantly different (*p* < 0.05).

### Parallel Indirect Effects for Full Sample

In regards to aim 1, results from the parallel indirect effects path model are shown in Figure 1, and bias-corrected bootstrap results for indirect effects are reported in Table 3. The only feeding practice that had a significant indirect effect was restrictive feeding (standardized effect = 0.03). The bootstrapped unstandardized indirect effect was 0.03 [CI (0.01, 0.06), *p* < 0.05]. Parent emotional eating was associated with higher levels of restrictive feeding, β = 0.16, *p* < 0.05, and higher levels of restrictive feeding were associated with greater parent-reported child emotional eating, β = 0.19, *p* < 0.05. The direct effect from parent emotional eating to child emotional eating was still significant after accounting for the indirect effect of restrictive feeding [*estimate* = 0.23, *p* < 0.001, CI (0.13, 0.31)], suggesting that the indirect effect of restrictive feeding only partially accounts for the association between parent and child emotional eating. Although no other indirect effects were statistically significant, parent emotional eating was also significantly and positively associated with instrumental feeding (β = 0.13, *p* < 0.05), and parent emotion regulation feeding was significantly and positively associated with parent-reported child emotional eating, β = 0.32, *p* < 0.001. Additionally, the total effect of parent emotion eating on child emotional eating was significant [*estimate* = 0.30, *p* < 0.001, CI (0.18, 0.38)].

**TABLE 3:** Results for parallel indirect effects of parental feeding practices (*N* = 410).

Feeding practice (CFPQ)	Effect	*SE*	95% CI
Emotion regulation	0.03 (0.03)	0.02	[−0.01, 0.06]
Instrumental	0.01 (0.01)	0.01	[−0.00, 0.03]
Restrictive	0.03 (0.03)	0.01	[0.01, 0.06]

*CFPQ, Comprehensive Feeding Practices Questionnaire; *SE*, standard error; CI, confidence interval. Standardized coefficients are presented for indirect effects. Bias-corrected bootstrapped, unstandardized indirect effects are reported in parentheses. Bootstrapped standard errors and confidence intervals are presented (bootstrap sample = 10,000).*

### Moderated Indirect Effects by Parent Gender

The exploratory results for aim 2 indicate that these associations are conditional on parent gender and should be interpreted differently for mothers compared to fathers. Specifically, results for the moderated indirect effect models showed that there were significant differences in indirect effects by parent gender. The test of nested model comparisons (see Table 4) revealed that there was a significant decrement in fit when constraining model paths as equal (model 2), indicating that allowing parameters to differ for mothers and fathers (model 1) was a better model (χ^2^_diff_ = 56.75, Δ*df* = 12, *p* < 0.001). There was no significant change in model fit between the model with constrained model paths and intercepts (model 3) compared to constrained model paths only (model 2). An additional chi-square difference test was conducted to directly compare the model with constrained paths and intercepts and the model with freely estimated parameters for mothers and fathers. Results showed that the model with freely estimated parameters fit the data significantly better than the constrained model (χ^2^_diff_ = 61.47, Δ*df* = 17, *p* < 0.001), indicating that both model paths and intercepts should be freely estimated for mothers and fathers.

**TABLE 4:** Model comparison results for conditional indirect effects.

Model	*df*	χ^2^	χ^2^_diff_	*p*
1. Free parameters	0	0		
2. Constrained paths	12	56.75	56.75	<0.001
3. Constrained paths and intercepts	17	61.47	4.72	0.47

*df, degrees of freedom; χ^2^, chi-square statistic; χ^2^*_diff_*, chi-square difference; *p*, *p*-value.*

**FIGURE 2:**
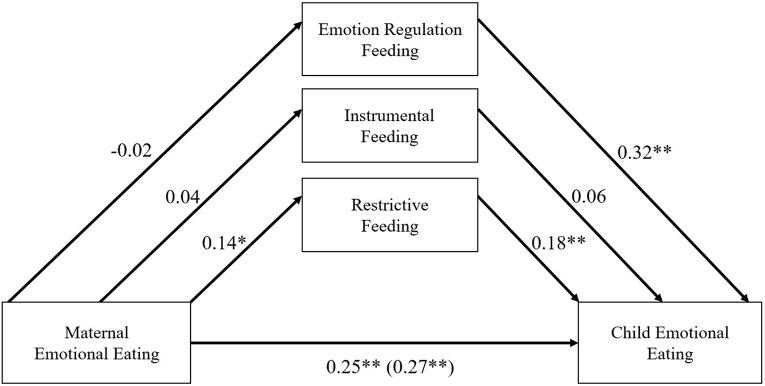
Standardized regression estimates for mother’s conditional effects. *p < 0.05, **p < 0.001. Covariates included parent stress, parent negative affect, child age, and child gender on all model paths.

**FIGURE 3:**
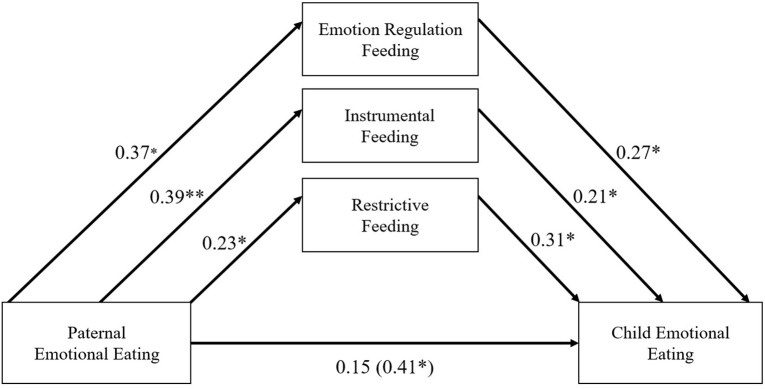
Standardized regression estimates for father’s conditional effects. *p < 0.05, **p < 0.001. Covariates included parent stress, parent negative affect, child age, and child gender on all model paths.

Estimates from the moderated parallel indirect effects path model are presented separately for mothers (see Figure 2) and fathers (see Figure 3). All indirect effects are reported in Table 5. There was evidence of a moderated indirect effect of feeding practices between parent and child emotional eating for both mothers and fathers. For mothers, the only significant indirect effect was for restrictive feeding (standardized effect = 0.03). The bootstrapped unstandardized indirect effect was 0.02 [CI (<0.01, 0.05), *p* < 0.05]. Mothers who reported greater emotional eating also reported greater use of restrictive feeding practices (β = 0.14, *p* < 0.05); higher levels of restrictive feeding in mothers was also associated with significantly greater parent-reported child emotional eating (β = 0.18, *p* < 0.001). The direct effect between maternal and child emotional eating was still significant [*estimate* = 0.25, *p* < 0.001, CI (0.14, 0.33)] suggesting that the indirect effect of restrictive feeding only partially accounts for the association between maternal and child emotional eating. There were no other significant indirect effects for mothers. However, there was a significant, positive association between maternal emotion regulation feeding and parent-reported child emotional eating (β = 0.32, *p* < 0.001). The total effect of maternal emotion eating on child emotional eating was also significant [*estimate* = 0.27, *p* < 0.001, CI (0.15, 0.36)].

**TABLE 5:** Results for conditional indirect effects by parent sex.

	Fathers (*N* = 86)			Mothers (*N* = 324)		
Feeding practice (CFPQ)	Effect	*SE*	95% CI	Effect	*SE*	95% CI
Emotion regulation	0.10 (0.09)	0.05	[0.01, 0.19]	−0.01 (−0.01)	0.02	[−0.04, 0.02]
Instrumental	0.08 (0.08)	0.04	[0.01, 0.16]	<0.01 (<0.01)	0.01	[−0.00, 0.01]
Restrictive	0.07 (0.06)	0.04	[0.01, 0.15]	0.03 (0.02)	0.01	[<0.01, 0.05]

*CFPQ, Comprehensive Feeding Practices Questionnaire; *SE*, standard error; CI, confidence interval. Standardized coefficients are reported for indirect effects. Bias-corrected bootstrapped, unstandardized indirect effects are reported in parentheses. Bootstrapped standard errors and confidence intervals are presented (bootstrap sample = 10,000).*

For fathers, there were significant indirect effects for emotion regulation (standardized effect = 0.10), instrumental (standardized effect = 0.08), and restrictive (standardized effect = 0.07) feeding practices. The bootstrapped unstandardized indirect effects were 0.09 [CI (0.01, 0.19), *p* < 0.05], 0.08 [CI (0.01, 0.16), *p* < 0.05], and 0.06 [CI (0.01, 0.15), *p* < 0.05], respectively. Fathers with high levels of emotional eating reported greater use of emotion regulation (β = 0.37, *p* < 0.001), instrumental (β = 0.39, *p* < 0.001), and restrictive (β = 0.23, *p* < 0.001) feeding practices. In turn, greater use of emotion regulation, instrumental, and restrictive feeding practices in fathers was associated with significantly greater parent-reported child emotional eating (β = 0.27, β = 0.21, β = 0.31, respectively, *p* < 0.05). Further, the direct effect for fathers was no longer significant after accounting for these indirect effects [*estimate* = 0.15, *p* = 0.17, CI (−0.06, 0.33)] indicating that these indirect effects through paternal feeding practices fully account for the association between paternal and child emotional eating. The total effect of paternal emotion eating on child emotional eating was statistically significant [*estimate* = 0.41, *p* < 0.05, CI (0.15, 0.57)].

## Discussion

### Parent-Child Emotional Eating and Feeding Practices in the Full Sample

The current study examined the role of various feeding practices in the association between parent emotional eating and child emotional eating, and explored how the indirect effect of these feeding practices differed according to parent gender. This study builds upon the previous literature by being the first to simultaneously compare the indirect effects of these common parental feeding practices in the association between parent and child emotional eating during middle childhood. Further, results from the current study can be used to inform future parenting interventions aimed at reducing child emotional eating behaviors by documenting important differences between mothers and fathers in the influence of parent emotional eating and feeding practices on child emotional eating.

In the full sample, including both mothers and fathers, results from aim 1 suggest the positive association between parent emotional eating and child emotional eating may be partially explained by higher levels of restrictive feeding, above and beyond the effect of emotion regulation and instrumental feeding practices, which have also been found to relate to both parent and child emotional eating separately (e.g., Rodgers et al., 2014; Powell et al., 2017). However, follow up analyses revealed that results differed by parent gender, suggesting interpretations of the influence of parental feeding practices in the association between parent and child emotional eating should occur separately for mothers and fathers. Specifically, results from exploratory aim 2 showed that the role of parental feeding practices in the association between parent-child emotional eating does vary by parent gender. These results provide initial evidence that there may be different models of how parent emotional eating and feeding influence child emotional eating for mothers and fathers, and highlights the importance of continuing to explore differences in parent-child eating and feeding behaviors by parent gender in future investigations.

### Maternal Emotional Eating and Feeding Practices on Child Emotional Eating

The results for mothers are similar to the results from the first aim with the combined sample of mothers and fathers. This is likely because mothers are overrepresented in the full sample which biases the results toward the effects for mothers. Among mothers, greater restriction of children’s food intake partially accounted for the positive association between maternal emotional eating and child emotional eating, above and beyond the effect of emotion regulation and instrumental feeding. Mothers who engaged in higher levels of emotional eating were more likely to use restrictive feeding practices; greater restrictive feeding was then positively associated with higher levels of emotional eating in their children. There were no other significant indirect effects of feeding practices in the association between mother-child emotional eating. This is in contrast with previous research suggesting emotion regulation feeding does significantly influence the association between parent and child emotional eating (Rodgers et al., 2014; Tan and Holub, 2015), and studies that document significant associations between instrumental feeding, and maternal emotional eating (Wardle et al., 2002) and child emotional eating (Rodgers et al., 2013, 2014).

These contradictory results could be due to differences in child age among samples. For example, many studies investigating mother-child emotional eating behaviors include mothers of preschool-aged children or younger (e.g., Wardle et al., 2002; Rodgers et al., 2014), whereas the current study aimed to examine parent emotional eating, feeding practices, and child emotional eating during middle childhood. It may be that parent feeding practices differentially influence the relation between parent and child emotional eating based on the developmental stage of the child. In a study of children ages 2–10 years-old, researchers failed to find a significant indirect effect of restrictive feeding due to a non-significant effect between maternal emotional eating and restrictive feeding practices, however, there was still a significant association between maternal use of restriction and child emotional eating (Kröller et al., 2013). The current sample included parents of school-aged children (ages 5–13) in which there was a significant, positive correlation between maternal emotional eating and restrictive feeding. It is theoretically possible that the association between maternal emotional eating and restrictive feeding strengthens as their children get older, especially if their children are openly engaging in emotional eating. If mothers are repeatedly observing their children overeat in emotional situations, they may be more likely to attempt to restrict their food consumption due to concerns about weight gain. More use of maternal restriction, in turn, can increase children’s consumption of restricted foods which are more likely to be energy-dense foods (Fisher and Birch, 1999) and interfere with children’s awareness of internal hunger cues (Jansen et al., 2012). This increased preference toward energy-dense foods is also associated with higher levels of emotional eating in children (Nguyen-Michel et al., 2007). More research is necessary to elucidate how different periods of child development and other mechanism influence the association between maternal emotional eating, feeding practices, and child emotional eating. Future studies should aim to explore how these associations differ for children of different ages and reproduce these results in longitudinal studies that can determine the direction of these effects.

Although there was a significant indirect effect of restrictive feeding, greater maternal emotional eating was still directly associated with higher levels of child emotional eating, even after accounting for maternal use of all three feeding practices. This direct effect was not significant for fathers. Numerous studies have documented a direct association between mother and child emotional eating (e.g., Elfhag et al., 2010; Kröller et al., 2013; Rodgers et al., 2014), with one finding that mothers’ emotional eating had a stronger influence on child emotional eating compared to fathers’ (Snoek et al., 2007). Previous literature suggests that this direct effect from mother to child emotional eating could be explained by modeling (Morrison et al., 2013); children who observe their mothers eating in response to emotional stimuli may do the same when they experience emotional cues themselves. There is evidence to suggest that mothers are more likely to take primary responsibility for feeding their child (Blissett et al., 2006), so it is possible that this effect of maternal modeling is due to mothers spending more time with their child. However, other studies have shown that fathers are now spending more time with their children (Feinberg, 2003) and reporting more responsibility for feeding their child (Mallan et al., 2014) than in the past. Since traditional gender roles and responsibilities have evolved in recent decades (see Bianchi, 2000), mothers may no longer be spending more time with their child or taking primary responsibility for feeding compared to fathers. Thus, there may be alternative factors that can explain the direct effect between mother-child emotional eating that was not present for fathers. In particular, previous research has demonstrated that the direct effect between maternal and child emotional eating may be influenced by maternal psychopathology. Extant studies show that child emotional eating is influenced by maternal anxiety and distress (Kidwell et al., 2017), maternal attachment anxiety (Hardman et al., 2016), and maternal depression, anxiety, and stress (Rodgers et al., 2014). While the current study controlled for maternal negative affect and stress in path models, other aspects of maternal psychopathology may explain the direct effect between maternal and child emotional eating. Further, in the current study, maternal emotion regulation feeding was positively associated with child emotional eating even though the indirect effect was non-significant. Thus, a reciprocal or bidirectional effect is possible, such that mothers use emotion regulation feeding to regulate their child’s emotions because they have observed their child using food to cope with their emotions and believe it is an effective coping strategy, instead of or in addition to mothers’ own emotional eating driving the use of emotion regulation feeding. Future research in this area should aim to examine mechanisms that could explain the link between mother and child emotional eating (e.g., maternal psychopathology, feeding responsibility), and reproduce this direct effect between maternal and child emotional eating in longitudinal studies with multi-method assessments to fully understand how these processes operate.

### Paternal Emotional Eating and Feeding Practices on Child Emotional Eating

In contrast to the results for mothers, there were significant indirect effects between fathers’ emotional eating and their child’s emotional eating through all three feeding practices. Paternal emotion regulation, instrumental, and restrictive feeding practices fully accounted for the association between father and children emotional eating. Results from the current study show that fathers with higher levels of emotional eating were more likely to report greater use of emotion regulation feeding, which was associated with higher levels of emotional eating in their children. Emotional eating can operate as a successful, although maladaptive, coping mechanism for adults (Spinosa et al., 2019). Thus, fathers who engage in high levels of emotional eating may be more likely to use food to regulate their child’s emotional arousal because they believe it is an effective coping strategy and can be used as a simple way to reduce their child’s negative emotions immediately. There is some literature to support this indirect effect of emotion regulation feeding between paternal and child emotional eating. In a study of mothers and fathers, researchers reported that emotion regulation feeding practices mediate the association between parent and child emotional eating, but only when children have low self-regulation of eating (Tan and Holub, 2015). Thus, there are likely other factors involved that could further explain the connections among paternal emotional eating, emotion regulation feeding, and child emotional eating. This is a great direction for future research as there are currently limited studies investigating these processes in samples of fathers or examining how these factors differ for mothers compared to fathers in mixed samples.

In the current study, fathers reported significantly greater use of instrumental feeding practices compared to mothers. The results for fathers also demonstrate that the tendency to use food as a means of reinforcing specific children’s behaviors (i.e., instrumental feeding) accounts for the association between paternal and child emotional eating. This finding provides initial evidence that using food as a reward or punishment may explain the link between father and child emotional eating. Similar to emotion regulation feeding, fathers who use food to cope with their own negative emotions (i.e., emotional eating) might be more likely to use instrumental feeding practices because they believe food will serve as positive reinforcement. Thus, fathers may use food to regulate their child’s behavior since it is an easy and accessible way to solve short-term behavior difficulties (i.e., effective behavior management strategy). It is also possible that fathers use more instrumental feeding practices because they do not perceive their children to be able to successfully regulate their own behaviors. In support of this, previous studies have shown that fathers report lower self-regulation of eating scores for their children compared to mothers (Powell et al., 2017). There may also be child factors involved in the relation among paternal emotional eating, instrumental feeding, and child emotional eating. For example, researchers have suggested that children’s own eating self-regulation abilities could explain the link between paternal, and maternal, use of instrumental feeding and child emotional eating (Powell et al., 2017). Since studies have documented significant conditional (Tan and Holub, 2015) and indirect effects (Powell et al., 2017) related to child self-regulation of eating in models of parent-child emotional eating and feeding, further investigations on father-child emotional eating and feeding practices should explore both how parent perceptions and child eating self-regulation abilities influence these processes.

The last significant indirect effect among fathers was for restrictive feeding practices. Similar to the results for mothers, fathers who reported higher levels of emotional eating were more likely to report higher levels of restrictive feeding and emotional eating of their child. However, fathers did report significantly greater use of restrictive feeding practices compared to mothers, and the effect sizes (i.e., standardized path coefficients) among fathers’ emotional eating, restrictive feeding, and child emotional eating were higher than those for mothers. This is in line with previous research documenting that fathers are more likely than mothers to use restrictive feeding due to weight-related reasons (Musher-Eizenman et al., 2007). These results are among the first to document a significant indirect effect of restrictive feeding practices between paternal and child emotional eating. There is literature to suggest that paternal body dissatisfaction is related to greater control of children’s food intake (Blissett et al., 2006). Thus, it is reasonable to assume that the association between paternal emotional eating and restrictive feeding could be explained by father’s own sense of eating attitudes and body image beliefs. For example, fathers who engage in emotional eating and are dissatisfied with their appearance may want to restrict their child’s food intake because they are trying to prevent their child from gaining weight and developing similar body dissatisfaction beliefs. This restrictive feeding can lead to increased consumption of high-calorie foods in children (Boots et al., 2015), which is another risk factor for emotional eating in children (Nguyen-Michel et al., 2007). Recent research has also shown that emotion dysregulation can moderate the relation between emotional eating and disordered eating symptoms (e.g., dietary restraint, concerns about eating, shape, or weight) in adults (Barnhart et al., 2021), suggesting that improving parents’ emotion regulation skills may weaken the relation between their emotional eating and use of restrictive feeding practices, and subsequently reduce emotional eating in their children. Future studies should aim to identify additional mechanisms (e.g., paternal body image beliefs and emotion regulation, child caloric intake) that could explain the links between paternal emotional eating, restrictive feeding, and child emotional eating.

Overall, the conditional indirect effects for fathers suggest that the use of food to regulate child behavior (i.e., instrumental feeding) or emotions (i.e., emotion regulation feeding) may be more salient for fathers, compared to mothers, when it comes to their child’s emotional eating. The present study advances the literature by documenting indirect effects of emotion regulation, instrumental, and restrictive feeding in the association between father’s own and their child’s emotional eating. Further research is needed to reproduce these findings and further examine the roles of different feeding practices between parent-child emotional eating, especially in fathers. Specifically, future studies in this area should focus on reproducing these exploratory results in balanced parent samples that include both mothers and fathers or samples of only fathers. Additional work could build upon the current results by investigating mechanisms that link paternal emotional eating to feeding practices (e.g., emotion regulation, body dissatisfaction) or those that account for the association between father’s feeding practices and their child’s emotional eating (e.g., child self-regulation, caloric intake, coping skills).

## Limitations

There are several limitations of the current study that should be noted. First, this study was cross-sectional in nature and utilized all parent-report measures. Thus, it is not possible to determine directionality of any findings. Some researchers have found evidence that parental feeding practices predict later child emotional eating and not the other way around (Steinsbekk et al., 2016). Alternatively, others have argued that the relation between feeding and child emotional eating is bi-directional (Faith et al., 2004), particularly during middle childhood (i.e., children ages 6–10 years; Steinsbekk et al., 2018). Future work in this area should include longitudinal designs with multi-method assessments (e.g., collecting data from multiple informants, in different settings, with a variety of measures) to better understand the direct and indirect effects among parent emotional eating, feeding practices, and child emotional eating. Second, there were many more mothers included in this study compared to fathers. Unbalanced sample sizes can lead to underestimated moderation effects (Stone-Romero and Anderson, 1994). This is less of an issue for the current findings, since results still showed significant moderation effects; however, the exact parameter values could still be underestimated, especially for fathers’ indirect effects since there were fewer fathers included in the sample compared to mothers. Third, although previous research has found differences in emotional eating and feeding based on child gender, the current study did not model differences by child gender or parent gender by child gender dynamics as this was not the focus of the study and would likely be underpowered to do so. Fourth, the parent stress measure included as a covariate in this study was only a single, subjective question of general stress rather than a specific parenting stress index as it was the only stress measure available. Future studies should include a more comprehensive assessment of parenting stress that may be more salient for parent emotional eating, feeding and child emotional eating, such as the Parenting Stress Index (Abidin, 1983) or Caregiver Strain Questionnaire (Brannan et al., 1997; see Holly et al., 2019 for more). One last limitation to note is that this was a convenience sample. As a result, the sample included mostly non-Hispanic White participants and no underrepresented gender identities, which limits the generalizability of the results and is not representative the diversity of genders, including 11% of the United States population that doesn’t identify with the binary (Wilson and Meyer, 2021). Further, the measure of parent and child gender included in this study was also limited as it did not include the full continuum of gender identities which may have influenced participants to choose a binary option.

## Conclusion

In sum, the current study contributes to the literature by highlighting putative mechanisms that could explain the relation between parent-child emotional eating behaviors and providing preliminary evidence that these mechanisms should be examined and interpreted differently for mothers and fathers. When considering both mothers and fathers simultaneously, restrictive feeding practices was the only feeding practice to partially account for the association between parent and child emotional eating. However, the current results suggest that patterns of parent emotional eating, feeding, and child emotional eating operate differently when considering the influence of maternal and paternal behaviors separately. Maternal use of restrictive feeding partially accounted for the relation between maternal and child emotional eating, but maternal emotional eating still was significantly associated with child emotional eating. In contrast, paternal use of emotion regulation, instrumental, and restrictive feeding practices fully accounted for the association between father-child emotional eating. Taken together, results suggest that restrictive feeding practices may be a key mechanism between parent and child emotional eating for mothers and fathers. These results also suggest that there is a strong direct effect between mother-child emotional eating, but an indirect effect of father’s regulation skills (i.e., more use of food to regulate behavior and emotions of their child) between father-child emotional eating. The current study provides initial evidence that fathers’ feeding practices may be more salient than mothers’ when it comes to the intergenerational transmission of emotional eating. As such, research and intervention efforts aimed at reducing emotional eating in children may benefit from focusing on father’s feeding practices (e.g., emotion regulation, instrumental, and restrictive feeding practices), and mother’s own emotional eating behaviors, rather than parental feeding practices in general.

## Data Availability Statement

The dataset and analysis script generated for this study can be found in the Open Science Framework Repository for this study at the following link: https://osf.io/muzbj/.

## Ethics Statement

The studies involving human participants were reviewed and approved by Institutional Review Board of the University of Oregon. The patients/participants provided their written informed consent to participate in this study.

## Author Contributions

NK, EB, and NG designed the study on which the present analyses were based. EB and NG collected the data. ST analyzed the data and wrote the manuscript. All authors edited drafts and approved the final version.

## Conflict of Interest

The authors declare that the research was conducted in the absence of any commercial or financial relationships that could be construed as a potential conflict of interest.

## Publisher’s Note

All claims expressed in this article are solely those of the authors and do not necessarily represent those of their affiliated organizations, or those of the publisher, the editors and the reviewers. Any product that may be evaluated in this article, or claim that may be made by its manufacturer, is not guaranteed or endorsed by the publisher.
